# Exploring and explaining low participation in physical activity among children and young people with asthma: a review

**DOI:** 10.1186/1471-2296-9-40

**Published:** 2008-06-30

**Authors:** Brian Williams, Alison Powell, Gaylor Hoskins, Ron Neville

**Affiliations:** 1 Social Dimensions of Health Institute, University of Dundee, Dundee, UK; 2 General Practice Section, Division of Community Health Sciences, University of Edinburgh, UK; 3 Division of Community Health Sciences, University of Dundee, Dundee, UK; 4 Division of Community Health Sciences, University of Edinburgh, Edinburgh, UK

## Abstract

**Background:**

Asthma is the most common chronic illness among children and accounts for 1 in 5 of all child GP consultations. This paper reviews and discusses recent literature outlining the growing problem of physical inactivity among young people with asthma and explores the psychosocial dimensions that may explain inactivity levels and potentially relevant interventions and strategies, and the principles that should underpin them.

**Methods:**

A narrative review based on an extensive and documented search of search of CinAHL, Embase, Medline, PsycINFO and the Cochrane Library.

**Results & Discussion:**

Children and young people with asthma are generally less active than their non-asthmatic peers. Reduced participation may be influenced by organisational policies, family illness beliefs and behaviours, health care advice, and inaccurate symptom perception and attribution. Schools can be reluctant to encourage children to take part in physical education or normal play activity due to misunderstanding and a lack of clear corporate guidance. Families may accept a child's low level of activity if it is perceived that breathlessness or the need to take extra inhalers is harmful. Many young people themselves appear to accept sub-optimal control of symptoms and frequently misinterpret healthy shortness of breath on exercising with the symptoms of an impending asthma attack.

**Conclusion:**

A multi-faceted approach is needed to translate the rhetoric of increasing activity levels in young people to the reality of improved fitness. Physical activity leading to improved fitness should become part of a goal orientated management strategy by schools, families, health care professionals and individuals. Exercise induced asthma should be regarded as a marker of poor control and a need to increase fitness rather as an excuse for inactivity. Individuals' perceptual accuracy deserves further research attention.

## Background

Asthma in children and young people is a major health problem [1-4]. For reasons that are not fully understood the incidence and prevalence of asthma have increased considerably over the last thirty years, particularly for children and young people [5,6]. Despite a reduction in incidence since it peaked in 1993 the UK asthma rate continues to be six times higher among children now than it was in 1976 [3,7]. The prevalence rate in the 2–15 years age group is reportedly one fifth of the population (20.7%) and when compared with 56 other countries the UK has one of the highest prevalence rates of wheeze and asthma in 13–14 year old children [5].

The burden on health services from asthma is extensive and increasing [5]. At primary care level asthma accounts for 1 in 5 of all child GP consultations [8], while within secondary care in England and Wales in 1999, there were over 30,000 asthma related hospital admissions and 25 child deaths [7]. Asthma is thus a common condition affecting a substantial and increasing proportion of children and young people [3] and as a result provides a challenge in management for the individual sufferer and for health services as a whole.

In addition to the direct effect the condition has on the wellbeing of an individual, it is increasingly recognised that the health of sufferers can be further compromised because of the impact that asthma may have on their participation in physical activity [6,9]. This paper presents a narrative synthesis based on a scoping review of the literature in order to provide an overview of evidence and argument in this area, and therefore inform decisions about the future direction of empirical studies and/or more specifically focussed systematic reviews[10,11]. It reviews and discusses recent literature outlining the growing problem of physical inactivity among young people with asthma and explores the psychosocial dimensions that may explain inactivity levels and potentially relevant interventions and strategies, and the principles that should underpin them.

## Methods

The aim to provide an overview of evidence and argument in order to direct future research enquiry and inform potential policy indicated that a "scoping review" approach was required. Systematic reviews require a small number of highly specific questions and thus presuppose a pre-existing knowledge and understanding of the importance and priorities of key topics and issues. Scoping reviews are designed to identify and provide a rationale for these issues[11], and have been used in a wide number of clinical areas [12-14]. They do not, therefore, claim to provide an exhaustive account of the evidence base for all specific questions.

Given the breadth of literature relating to both asthma and physical activity, and the changing characteristics of each, it was decided to initially identify relevant review articles within the recent literature, and then use these to identify other key empirical pieces. We searched the Cumulative Index of Nursing and Allied Health Literature (CinAHL); Excerpta Medica Database (Embase); Medlars Online (Medline); Psychological literature (PsycINFO); and the Cochrane Library for relevant studies published between 2001–2005 inclusive (see Table 1). Abstracts were read independently by two researchers (BW and AZ – see acknowledgements) and the decision to include them compared. Disagreements were addressed through discussion of the evidence and rationale for each paper. In accordance with scoping review principles, and in order to facilitate subsequent identification of yet unknown key issues and arguments within the literature, selection criteria were purposefully broad. Papers whose central focus was not asthma were excluded at this stage as were non-empirical papers (e.g. commentaries, editorials, and discussion pieces). Sixty-one papers were identified as relevant and full papers obtained. The review articles identified by these searches allowed us to map the broad issues around which the remainder of this paper is structured. We then used the reviews, alongside key papers cited within them (which we obtained) in order to inform the narrative synthesis of arguments, issues and debates required.

**Table 1:** Initial Scoping Search Strategy and Results.

	**Search (in title, abstract key word)**	**Result**
1	"physical activity" or "Physical activities" or "exercise or "sport"	322,076
2	"child" or "children" or "young people" or "teenager" or teenagers" or "young women" or young men"	2,080,581
3	"asthma" or "chronic illness" or "chronic disease"	354, 750
4	1 AND 2 AND 3	3,125
5	Remove duplicates	2,392
6	Limit to English Language	1860
7	Limit to "review articles"	611
8	Limit year 2001–2005	168
	Papers identified from Cochrane library	3
	Full papers obtained	61

## Results & Discussion

The literature demonstrated empirical evidence documenting the importance of physical activity for children with asthma, reduced levels of participation, social and psychological factors influencing low participation, and principles that should underpin strategies to increase physical activity among children and young people with asthma. These are discussed in turn below.

### The importance of physical activity for children and young people with asthma

Physical activity is generally accepted to be of advantage to young children in terms of bone development, motor skills, improved cardiovascular fitness, and self esteem [15,16]. Physical activity is also important for children and young people with asthma. Several studies have identified significant improvements in aerobic fitness [17,18] and asthma-related benefits such as reduced hospital admissions, reduced absenteeism from school, fewer consultations with health professionals, reduced medication use [19], and improved ability to cope with asthma [18]. In addition, it remains clear that being able to participate in physical activity, particularly at school, is an important contributing factor for psychological wellbeing by, for example, reducing the body dissatisfaction that can be associated with asthma [1,20,21]. Furthermore, studies involving dietary alteration have shown that weight loss for obese people with asthma can improve their asthma symptoms. [22]. It is therefore feasible that physical activity that is conducive to weight loss might be helpful for obese children and young people with asthma.

In addition to the above benefits physical activity may also help protect against the potential increased risk of osteoporosis associated by the prolonged steroid therapy that some children and young people with asthma may experience [23]. Although direct evidence of such benefits is currently lacking for asthma, evidence exists to show that weight-bearing physical activity can increase bone mineral content and reduce osteoporosis risk later in life among children with acute lymphoblastic leukaemia [15].

Despite these varied benefits the majority of studies of physical activity training programmes for children and young people with asthma have shown no change in actual baseline lung function [24,25] or in the occurrence or degree of exercise-induced asthma [19]. Further RCTs and systematic reviews on the effects of physical training need are required [17].

Because of this range of physical, psychological and social benefits, current evidence suggests that children and adolescents with asthma should be encouraged to participate in regular physical activity. This may improve asthma management and general health and minimise the generic risks associated with low levels of physical activity [1,19].

Although the existence of a respiratory condition might be expected to prevent engagement in such activities the overwhelming majority of studies show that people with asthma *can *exercise safely if medicated appropriately and can significantly improve their cardiovascular fitness and quality of life by doing so [6]. Indeed, a substantial proportion of sportsmen and women who compete at the elite international level have a diagnosis of asthma [26] or experience exercise-induced asthma [9]. The consensus of many authors is therefore that inactivity or reduced activity in the presence of an asthma diagnosis should not be accepted [9]. Instead, an exercise 'prescription' should be part of the management plan for all people with asthma [6].

### Evidence for reduced participation in physical activity among children and young people with asthma

Despite the importance of physical activity to children and young people with asthma, and although some studies have failed to demonstrate a significant difference in physical activity levels between asthmatic and non-asthmatic children[27], there is growing evidence that they are in fact *less *active than their non-asthmatic peers. Lack of participation in physical activity by children and young people with asthma is unsurprising given that physical activity levels are known to be falling among young people in most industrialised nations [28,29]. These rates fall even further as children reach adolescence [16], particularly in teenage girls. At secondary school age, boys report more physical activity at 11, 13 and 15 than girls and are more likely to meet the guideline of 60 minutes moderate activity per day [30].

Although the majority of evidence points to reduced physical activity among young people with asthma, conceptual and methodological problems, particularly in relation to confounding, has resulted in contradictory evidence [31]. These contradictions arise partly as a result of methodological and disease detection/diagnostic issues. Engagement in significant levels of physical activity can lead to improved detection; particularly exercise induced asthma (EIA). Consequently, activity rates in children and young people with asthma may appear artificially high because already-active children and young people are more likely to be diagnosed as having asthma.

Notwithstanding this methodological problem, studies in a range of countries suggest that children and young people with asthma *are *less active than their healthy peers and that they attribute their limited participation to their asthma. In a comparative study of 112 children with and without asthma Glazebrook et al found that children with asthma reported fewer physical activities than the non-asthma group (median 4 per day vs 6 per day). Furthermore, asthma was the strongest predictor of lower activity scores, followed by younger age[32]. In addition, a study of 137 United States (US) children aged 6–12 years found that those with asthma were less active than their peers in all classifications of activity level used [33]. In Germany, a study of 254 teachers in 46 schools across a range of ages found that only 60% of children with asthma took part in physical education lessons on the same basis as their healthy peers: the remaining 40% did not participate, or only participated sometimes or to a limited extent [34]. An Australian survey also found reduced activity and a belief that asthma was responsible. Parents reported that as a result of asthma 31% did not participate in sport; 21% did not ride a bicycle; 20% did not swim; and 18% did not take part in break time play at school [35]. Young people with asthma also believe that their physical activity is reduced as a result. A survey of 123,000 US young people aged 12–14 found that 52% of those with a diagnosis of asthma reported that their activities were curtailed because of their asthma [4].

A reduction in physical activity levels at the same time as an increase in asthma incidence and prevalence is a cause for concern and is likely to result in increasing numbers of people with asthma failing to attain their optimum health status and quality of life. The remainder of this paper explores the range of psychosocial factors that may explain low participation before briefly considering potentially relevant interventions and the principles that should underpin them.

### Factors affecting participation in physical activity by children and young people with asthma

Studies suggest that three inter-connected factors affect participation in physical activity by children and young people with asthma: the illness beliefs of young people [36-38]; parental and family beliefs [1,33]; and the knowledge and attitudes of teachers and the organisational arrangements in schools [39-42]. Underpinning all of these is the problem of interpretation of asthma symptoms [43-45]. Each of these issues is discussed in turn below. While availability of facilities might be assumed to be important as a predictor of engagement in activity the relationship is not clear cut as some studies fail to demonstrate a connection [33]. More detailed and focussed systematic reviews and meta-analyses of studies in this area are needed, particularly among young people with asthma.

#### Illness beliefs of young people with asthma

Many children and young people with asthma believe that limitations on their activity are an inevitable part of having asthma [36-38]. A study of 24 US teenagers with asthma showed that they perceived that despite their best efforts they were unable to participate to the level they wanted [38]. Consequently, there may be low levels of self-efficacy [46].

Some young people may find low self-efficacy in relation to engagement in physical activity as problematic, particularly where they and their peers value such engagement. In such circumstances motivation to engage in behaviour change would otherwise have been present [47,48]. In one UK study around 80% of secondary school pupils said that the resultant effect of not being able to participate in sport was the worst thing about having asthma and was potentially stigmatising, thus leading to a spoilt identity [37]. [49].

The desire to meet socially defined forms of normality or "ordinariness", thereby maintaining membership of a valued social group and avoiding stigma or social exclusion, is known to be important for young people [50-52]. Maintenance of such "normality or "ordinariness", particularly in relation to gender specific definitions, appears to be a potentially important factor affecting whether young people with asthma participate in physical activities. For example, a study of 24 US 14–18 year old asthma sufferers found that for boys, being normal involved participating in physical activity, and a desire not to be last in competitive games. This meant that some boys discounted what they knew about asthma management and pushed themselves too hard. For girls this normality was less likely to include activity. However, *both *boys and girls recognised that there was a risk of potential stigma and labelling and many did not want to be identified as having asthma [38].

Parents, teachers and coaches play important roles in providing normalising and gratifying opportunities for children with chronic illness who struggle to be competent and accepted by peers. However, many parents and teachers are concerned about the risks of physical activity in children and young people with asthma and instead deter them from activities [21].

#### Parental and family beliefs

The influence of family factors on the activities of young people is well documented For example, a survey of around 4000 US teenagers without asthma found that a range of factors including race, educational aspirations and maternal parenting style were associated with whether girls were more likely to engage in physical activity or to have sedentary leisure habits [53]. Teenage girls with higher academic rank or expectation and girls whose mothers had a parenting style that balanced responsiveness and control were more likely to have higher levels of activity and lower sedentary leisure habits. Putting a high value on health, appearance and achievement was associated with higher activity levels and reduced sedentary behaviour.

Against this wider background of family influence on young people, several studies of physical activity in children and young people with asthma suggest that parental and family beliefs play a key part in both enabling children and young people to manage their asthma effectively, and sometimes hindering effective management [40,54]. For example, a comparative study of exercise among asthmatic and non-asthmatic children, and their parents, found that more parents in the asthma group identified the child's health as a barrier to exercise (60.7% vs 11%)[32]. Similarly, a US focus group study of 47 parents of children with asthma found that their beliefs about asthma and its treatment led many to modify the asthma treatment plan set out by a health professional, and that many unnecessarily restricted their child's physical activity because of lack of information or misinterpretation of advice given. Other parents did not restrict activity as they were concerned about the psychological consequences of such restriction, but many were concerned that they were thereby increasing their child's symptoms [1]. In a study of low income *pre-school *children in the US, children who had a history of wheezing were found to be less active than their peers. This suggested that parents may consciously or unconsciously restrict their child's activities; it is possible that these attitudes persist once the child enters school [55]. For example, a study of 137 US children and parents found that the strongest predictor of high activity among 6–12 year olds was parental belief that exercise could help improve the child's asthma. Children whose parents believed that the child would become ill or upset from exercise were more likely to be inactive whereas children whose parents believed that they could do as much as their peers were more likely to be active [33].

There is evidence that parental concerns may stem, in part at least, from misperceptions about the nature, cause and cure of asthma itself. A study of Hong Kong parents of children with asthma identified a range of fears and misconceptions: they thought that asthma might be infectious; they were concerned that swimming and other forms of exercise would make it worse; and they believed that inhaled steroids should be avoided [56]. These studies underline the extent to which parental knowledge and beliefs about asthma in a range of countries may be founded on misconceptions and may hinder children's participation in physical activity. Problems may also arise when the perceptions of the young person with asthma differ markedly from those of parents or teachers. For example, a study of US teenagers found that some teenagers saw asthma as potentially dangerous but that teachers or family members often did not give credibility to their symptoms [38]. Another study found that carers judged asthma severity by one measure, the *frequency *of acute attacks, whereas for young people the impact that asthma had on their daily life and the extent to which they appeared different from their peers were more significant [36].

#### Knowledge and attitudes of teachers and organisational arrangements in schools

Surveys of teachers in a range of countries including the US, Germany, Hong Kong and the UK have consistently found low levels of knowledge and understanding of asthma, asthma and exercise and asthma management [1,34,57-59]. Although some teachers recognise that sport is important for children with asthma [57], many are unaware that medication may be taken before exercise to effectively prevent acute attacks and that children with asthma can be as competent as healthy children in sports [59]. Studies show that many teachers may hold outdated beliefs, for example that those with asthma should avoid PE lessons [59] or that asthmatic children usually have 'overprotective mothers' [57]. Many teachers acknowledge that they would like more information and training [60]: in one UK study of primary school teachers, 93% said that they did not know enough about asthma and 94% said that they had had no training on this issue [57]. Similarly high levels were seen in a US survey of 291 elementary school teachers in which 78% said they did not feel adequately prepared to teach children with a chronic illness, and 77% did not feel confident to deal with asthma [58]. Although 95% of primary school head teachers in one UK study reported that they were 'fairly confident' or 'very confident' about the care of pupils with asthma, two-thirds expressed concern about dealing with an asthma emergency [39].

Given the widespread evidence that school staff have a limited knowledge and understanding of asthma, it is likely that many are unaware of the importance and feasibility of physical activity for children and young people with asthma. Lack of knowledge about *how *to manage an asthma attack may lead to excessive caution and unnecessary restriction of activity. Even if individual teachers are aware that sport is important for pupils with asthma, the *organisational *arrangements may not support full participation by pupils with asthma. Studies in a range of countries suggest that care for children with asthma in schools is often disorganised [39-42]. For example, many schools do not keep adequate diagnostic registers and cannot therefore accurately identify pupils with asthma, many do not allow pupils to be responsible for their own inhalers and some require parents to administer inhalers [41]. Thus school policies may not facilitate appropriate asthma management around exercise and may contribute to teachers, children and parents opting for non-participation. Some reluctant exercisers may in turn take advantage of this lack of clear policies: teachers in some studies have reported that increasing numbers of children and young people are excusing themselves from activity by 'forgetting' their inhalers, bringing notes from parents or citing their doctor's advice as justification for their non-participation. This is unsurprising given that pupils' attitudes towards PE lessons are known to become less positive as children become older [16]. From the perspective of parents, many did not trust school staff (apart from school nurses) to supervise their child's asthma care and had more faith in the child or young person's ability to manage their own care [1] – faith that may be misplaced given the tendency of many parents to over-estimate the self management skills of adolescents [54]. This lack of trust in school staff may have implications for parents' willingness to accept school policies on participation in routine activities by pupils with asthma.

Central to the beliefs of children, parents and school staff about the relationship between asthma and physical activity is the problem of how ambiguous physical phenomena such as breathlessness are interpreted and attributed to either physical exertion or to asthma.

#### Symptom perception/interpretation

The literature outlined thus far suggests that child, parental and school beliefs about asthma are likely to influence engagement in physical activity. These beliefs are likely to originate both in a socially derived stock of knowledge about asthma and through past experience of asthma itself. Leventhal's self-regulation theory has suggested that such knowledge may be embodied in abstract and archetypal form [61]. This is then drawn upon at times of symptom interpretation by the sufferer or observer resulting in a cognitive interpretation of the symptoms being experienced (illness representations expressed as: identity/symptoms, timeline, cause, consequences and cure/control) and a parallel emotional reaction. Both these cognitive and emotional consequences are managed by engaging in coping behaviours [61,62] which are subsequently appraised and thus, potentially, used again. In the case of asthma this may be by avoiding physical activity or engaging in it depending on both underlying beliefs and past experience.

Consequently, how children, parents and teachers interpret signs such as breathlessness is likely to be at the heart of day to day management of asthma and physical activity. Misinterpretation and misattribution of signs may result in inappropriate coping strategies. For example, symptoms that may not be asthma-related (e.g. breathlessness due to physical exertion) may be misinterpreted as asthma resulting in exercise avoidance when in fact failure to engage in cardiovascular based activity is the cause. Similarly, if breathlessness is misattributed to lack of fitness when in fact it is asthma then young people may fail to use their medication appropriately and may continue exercising at levels of intensity that may prove problematic.

Although the *pathophysiology *underlying some asthma symptoms is characteristically distinct (e.g. wheeze) many studies show that children and parents do not find the *interpretation *of such symptoms to be straightforward [26,63] as meanings of wheeze may vary from epidemiological definitions[64]. A review of studies in paediatric asthma has revealed that children and adolescents with asthma show considerable variability in perceptual accuracy and frequently make clinically relevant errors that have the potential to affect self-management behaviour [43]. It is also clear from studies conducted in a range of countries that people of all ages tend to underestimate their asthma symptoms and overestimate their control [2,35,40,54]. This tendency may lead to under-medication and erratic adherence to treatment. Indeed, it has been estimated that adherence rates for asthma treatment range from as low as 30% to, at best, only 70% [65]. This means that at best many children and young people with asthma may be under-medicating themselves and settling for less exercise and a poorer quality of life than they could achieve by following recommended management strategies and treatment. At worst, a tendency to over-estimate asthma control may be life-threatening for some individuals: a study of children with asthma admitted to hospital suggests that children who present to hospital with hypoxia tend to perceive themselves as less breathless than non-hypoxic children [44].

The problem appears to be the interpretation of asthma symptoms. Dyspnoea (shortness of breath) is highly subjective and can occur independently of any actual constriction of the airways. Perception of dyspnoea may be influenced by asthma patho-physiology *and *by a range of social and psychological factors. Furthermore, there is an interrelationship between these factors as psychological factors such as stress have been shown to be sufficient to induce breathlessness in people with asthma [66]. In terms of patho-physiology, there is evidence to suggest that perception may vary depending on whether there is acute airway obstruction or prolonged airway obstruction. People with prolonged airway obstruction have been found to perceive symptoms less accurately than those with acute onset airway obstruction [67]. Greater asthma severity has been found to be associated with blunted symptom perception [68].

The range of social and psychological factors found to either influence, or be associated with asthma perception and interpretation includes age, gender, Body Mass Index (BMI), history of exercise-induced symptoms, and psychological state [68-71]. Perceptions of dyspnoea may be relatively precarious and easily swayed via these variables. For example, one study looked at the effect on dyspnoea reporting of giving children false feedback about their lung function (measured by peak flow) [72]. Children who were given a peak flow reading that was worse than the actual reading reported significantly more dyspnoea, while those who were given a reading that was considerably better than the true reading did not reduce their report of dyspnoea. In another study, children's perception of dyspnoea during resistive load breathing tasks decreased when they were shown film clips encouraging positive emotions [73]. No change occurred when the children were shown 'neutral' or 'negative' film clips. This suggests that psychological factors like emotional state can affect the perception of dyspnoea by children with asthma. This was borne out by another study in which children experiencing negative emotions were found to be *more *inclined to interpret exercise-related symptoms (e.g. fatigue, heart-pounding and sighing) as symptoms of airway obstruction [74]. Higher 'trait anxiety' has also been found to be associated with heightened symptom perception [68].

A review of symptom perception studies in asthma concluded that although more empirical data on psychological factors are required, studies to date do suggest that the perceptual accuracy *is *influenced by: competition between asthma and non-asthma sensory information; negative emotions; and acquired response tendencies (e.g. habituation to symptoms, repression of symptoms, selective perception, and false interpretation of symptoms) [45]. These factors may favour either blunted perception or over-perception. More research is needed on factors that affect the magnitude of dyspnoea reporting.

Given the potential for misinterpretation of symptoms a proportion of those children and young people diagnosed as having exercise-induced asthma (EIA) may not be experiencing asthma-related symptoms, but instead may be breathless on exercise due to a lack of cardiovascular fitness [26]. There may also be social reasons encouraging such misinterpretation. Siersted et al [75] have suggested that attributing breathlessness to EIA may be more acceptable to parents, coaches and children than the conclusion that the child is overweight or unfit. EIA is certainly very common [24,39]; it occurs in 70–80% of patients with persistent asthma and in 12–15% of the general population [76,77]. However, any over-attribution may have deleterious effects if the subsequent response of children and parents/teachers is to restrict physical activity rather than to encourage it (with or without appropriate pre-exercise asthma medication). This may mean children and young people miss out on the substantial fitness improvements that may result from appropriate exercise training, particularly in children with severe asthma [19].

There is, therefore, a paradox in that while *over-attribution *of breathlessness to asthma may deny children sporting opportunities *under-attribution *(in the form of under-diagnosis) of asthma symptoms may also cause children with undiagnosed asthma to struggle with untreated symptoms and thus fail to fulfil their sporting potential [26]. Untangling the causes of breathlessness and deciding on appropriate action is therefore a complex but important challenge for children and young people with asthma, teachers and health professionals.

### Principles and strategies to increase participation in physical activity

Addressing the multiple factors influencing participation in physical activity outlined in this paper is likely to be challenging and complex. An overview of contemporary evidence suggests that strategies must include: accurate detection, diagnosis and symptom management; acknowledge and address social and family contexts, be goal and value based and address the problem of symptom interpretation.

#### Detection, diagnosis and management

Increasing participation in physical activity by children and young people with asthma may require improvements in the wider field of asthma diagnosis and management. Although the health and social consequences of asthma-like symptoms in children with no diagnosis are substantial, there remains a risk of under-diagnosis of asthma, especially in girls, those with low physical activity and those with a high body mass [75]. For example, a survey of 123,000 US 12–14 yrs old found that 17% had asthma like symptoms but no diagnosis. Of those with no diagnosis 20% missed a half day of school or more a month because of wheeze and 25% limited their activities at least once a month [4]. In a study of a cohort of 7–9 year olds one in 20 children who had wheezed in the past 12 months reported a symptom profile consistent with unrecognised (and hence untreated) asthma [76]. Improved accuracy of initial diagnosis of asthma needs to be accompanied by appropriate ongoing management and review, not least because of the need for changes in management as the child matures. However, studies show that some children and young people with asthma are not being effectively managed: one study found that a third of children currently being treated for asthma in the UK were seen by a health professional less than once a year and that 25% had never had their lung function documented even with a peak flow meter [8]. The failure to perform lung function tests, coupled with the evident potential for exertional dyspnoea to be misinterpreted as asthma, suggests that it is likely that some children may be misdiagnosed as having asthma. It also suggests that studies of the prevalence of asthma that reply on self-report methods may *overestimate *prevalence. However, others studies have suggested that failure of parents to correctly interpret "wheeze" may lead to *underestimations *of prevalence within such surveys[78] thereby counteracting any potential misattribution in regard to exertional dyspnoea. Further research to more clearly assess the scale of such biases is required. The introduction of the Quality Outcomes Framework and associated financial payments to UK general practice may, however, reduce misdiagnosis, as has been evidenced in other clinical areas [79].

#### Acknowledging and addressing social and family contexts

Educating the individual with asthma about the feasibility and benefits of physical activity are unlikely to be effective if the family and school contexts are not also persuaded to encourage and facilitate participation [76]. A coherent asthma control strategy is likely to need to take into account the range of influences on people trying to manage their disease (e.g. family, friends, school peers, teachers) and to involve all of these stakeholders to ensure that the messages they deliver and the actions they take are mutually reinforcing [40]. Indeed a systematic review of interventions to promote physical activity among children and adolescents more generally found strong evidence that school based interventions with involvement of the family or community and multi-component interventions were effective in increasing physical activity [80]. It is likely that elements of such interventions, and the principles underlying their success, may be largely appropriate to young people with asthma as long as other more disease specific issues are simultaneously addressed. Parents, children and teachers need information about how and when those with asthma can participate in physical activity, about how to adjust medication before and during periods of extended activity and about the distinction between limitations at times of asthma exacerbation and overall limitations [1]. Such an approach may be both feasible and effective. For example, a US school asthma management intervention based on education for parents *and *children was shown at six months to reduce missed school days by two-thirds, to increase caregivers' perceptions of the amount of physical activity carried out pupils by 11% and to reduce daytime and night-time frequency of symptoms [81].

#### Goal and value based strategies

Strategies need to draw on existing research knowledge about 'what works' in increasing activity in individuals with chronic conditions. For example, studies show that information-only interventions may have a limited effect and that programmes that teach self-management skills may be more effective [40,82]. A survey of 38 children aged 8–10 years and their parents found that participation in activities increased significantly with increased asthma knowledge and the use of avoidance coping and support-seeking coping behaviours [83]. A study of adults with chronic illness found that a multi-component intervention model including collaborative identification of goals, identification of barriers, personally relevant problem-solving strategies and systematic follow-up and support was also effective in increasing activity [84]. Such programmes aim to foster 'self-competence' or self-efficacy so that the individual believes that they are capable of producing desired outcomes and avoiding negative outcomes [82,83]. This means that education and asthma management must take into account the patient's own self-management goals (as opposed to purely clinical objectives) [40].

Fostering self-efficacy will entail taking account of psychological factors and of differences between individuals (e.g. high self-regulators or low self-regulators). This suggests that physical activity may also need to be promoted in different ways to boys and girls. Strategies should probably take account of gender differences in regard to the relationship between self/social identity and physical activity, and the differing motivating factors underpinning involvement in sports and exercise [85]. However, while such targeting appears theoretically appropriate a recent review for children and young people more generally cast doubt on the evidence base to support the effectiveness for such differentiation [80]. Further research in this area is required. Despite this uncertainty, it continues to appear sensible to target individuals' key motivators irrespective of how they are socially patterned. Bailis et al [85] have suggested that some individuals may be more likely to participate if emphasis is placed on the availability of support while others may be motivated more by external challenges and targets. Similarly, arguments framed in terms of the health benefits of performing a behaviour have been shown to be more persuasive for some individuals than those framed in terms of the costs of non-performance [86].

#### Addressing symptom interpretation

Difficulties in interpreting asthma symptoms may be a major contributor to the beliefs held by young people with asthma, their parents and teachers. More research is needed to identify distinct types of perceptual errors, to identify factors related to perceptual inaccuracy, to examine the relationship between perceptual accuracy and relevant outcomes, and to identify effective interventions [43,44]. It is not yet possible to reliably predict which individuals are most at risk of making clinically serious errors. Health professionals need to adopt an individualized approach to symptoms to guide patient education and management; identify patients prone to making clinically relevant errors; and develop and implement interventions to improve accuracy of symptom perception, both for those who under-perceive their symptoms and those who over-perceive them [43]. Individuals who are prone to under-estimate their symptoms may benefit from training and feedback using peak flow meters in order to improve perceptual accuracy. Individuals who tend to make symptom magnification errors can be informed of which symptoms accurately predict their functioning and which do not, and can be helped to differentiate asthma-related sensations from those that are unrelated [43,44].

#### Strengths and limitations of the review

This breadth of topic and associated searching permitted within scoping reviews and associated narratives means that, as in this case, it is possible to "map" out the range of key contemporary issues on a given topic, and the linkages between them. It also provides the basis for decision making about areas which may be important to target for future primary research as well as those where more specifically defined systematic reviews may be needed. However, in achieving this there are unavoidable limitations. The breadth of topic and associated searches means that we cannot claim to have identified all relevant papers on each issue and have been forced to make judgments as to how deep and detailed investigations should be in relation to each topic area. Identification of one study and finding necessarily leads to a host of other underlying issues which could also be investigated and reported. However, we believe that we have successfully provided sufficient depth without sacrificing the breadth and interconnection of issues which are at the heart of a scoping type review. A scoping review cannot include a full quality appraise, weight or "pool" data[11], as in more meta-analysis procedures. However, we believe that these limitations do not interfere with the broader purposes and value of the scoping review and indeed provide the foundation for future systematic reviews in these areas.

## Conclusion

Asthma is a common problem which impacts on individuals, their families and society. The literature from many countries over several decades shows a clear pattern of short term barriers to increased activity preventing children and young people with asthma attaining long term physical fitness with all its consequent benefits to lung health, general health, social and psychological well-being. Activity and physical fitness deserve a place in goal setting for generic and individual asthma management plans. The way young people perceive and respond to physiological signals such as shortness of breath on exercise requires attention.

## Competing interests

The authors declare that they have no competing interests.

## Authors' contributions

BW conceived the review, collated the papers, reviewed and synthesised the findings and drafted the paper. AP, RN and GH reviewed the findings and contributed to the writing of the paper.

## Pre-publication history

The pre-publication history for this paper can be accessed here:



## References

[B1] Mansour ME, Lanphear BP, DeWitt TG (2000). Barriers to asthma care in urban children: parent perspectives. Pediatrics.

[B2] Rabe KF, Vermiere PA, Sorianio JB, Maier WC (2000). Clinical management of asthma in 1999: the Asthma Insights and Reality in Europe (AIRE) study. Eur Respir J.

[B3] Smyth RL (2002). Asthma: a major pediatric health issue. Respir Res.

[B4] Yeatts K, Sotir M, Music S, Herget C (2003). Health consequences for children with undiagnosed asthma-like symptoms. Arch Pediatr Adolesc Med.

[B5] ISAAC (1998). Worldwide variations in the prevalence of asthma symptoms: the International Study of Asthma and Allergies in Childhood (ISAAC). Eur Respir J.

[B6] Lucas SR, Platts-Mills TAE (2005). Physical activity and exercise in asthma: relevance to etiology and treatment. J Allergy Clin Immunol.

[B7] National Asthma Campaign (2001). Out in the open.  A true picture of asthma in the United Kingdom today. The Asthma Journal.

[B8] National Asthma Campaign (2002). An audit of children's asthma in the UK. The Asthma Journal.

[B9] Sheth KK (2003). Activity-induced asthma. Pediatr Clin N Am.

[B10] EPPI-Centre (2005). A scoping review of the evidence for incentive schemes to encourage positive health and other social behaviours in young people.

[B11] Arksey H, O'Malley L (2005). Scoping studies: towards a methodological framework. International Journal of Social Research Methodology: Theory & Practice.

[B12] Forbes C, Glanville J, Kleijnen J (2002). Psychological aspects of organ and tissue retention: a scoping review.

[B13] Kainth A, McDaid C, Glanville J, Wright K, Toon P, Forbes C (2003). Telephone consultations in primary care: A scoping review.

[B14] O'Malley L, Croucher K (2005). Housing and dementia care - a scoping of the literature. Health & Social Care in the Community.

[B15] White J, Flohr JA, Winter SS, Vener J, Feinauer LR, Ransdell LB (2005). Potential benefits of physical activity for children with acute lymphoblastic leukaemia. Pediatr Rehabil.

[B16] Trudeau F, Shephard RJ (2005). Contribution to school programmes to physical activity levels and attitudes in children and adults. Sports Med.

[B17] Ram FSF, Robinson SM, Black PN (2000). Effects of physical training in asthma: a systematic review. Br J Sports Med.

[B18] van Veldhoven NHMJ, Vermeer A, Bogaard JM, Hessels MGP, Wijnroks L, Colland VT, van Essen-Zandvliet EEM (2001). Children with asthma and physical exercise: effects of an exercise programme. Clin Rehabil.

[B19] Welsh L, Kemp JG, Roberts RGD (2005). Effects of physical conditioning on children and adolescents with asthma. Sports Med.

[B20] Kelsay K, Hazel NA, Wamboldt MZ (2005). Predictors of body dissatisfaction in boys and girls with asthma. J Pediatr Psychol.

[B21] Vitulano LA (2003). Psychosocial issues for children and adolescents with chronic illness: self-esteem, school functioning and sports participation. Child Adolesc Psychiatric Clin N Am.

[B22] Stenius-Aarniala B, Poussa T, Kvarnstrom J, Gronlund EL, Ylikahri M, Mustajoki P (2000). Immediate and long term effects of weight reduction in obese people with asthma: randomised controlled study. Britiah Medical Journal.

[B23] Villareal MS, Klaustermeyer WB, Hahn TJ, Gordon EH (1996). Osteoporosis in steroid-dependent asthma. Annals of Allergy Asthma & Immunology.

[B24] Carlsen KH, Carlsen KCL (2002). Exercise-induces asthma. Paediatr Respir Rev.

[B25] Strong WB, Malina RM, Blimkie CJR, Daniels SR, Dishman RK, Gutin B, Hergenroeder AC, Must A, Nixon PA, Pivarnik JM, Rowland T, Trost S, Trudeau F (2005). Evidence based physical activity for school-age youth. J Pediatr.

[B26] Orenstein DM (2002). Pulmonary problems and management concerns in youth sports. Pediatr Clin N Am.

[B27] Jones SE, Merkle SL, Fulton JE, Wheeler LS, Mannino DM (2006). Relationship between asthma, overweight, and physical activity among U.S. High School students. J Community Health.

[B28] Andersen LB, Harro M, Sardinha LB, Froberg K, Ekelund U, Brage S, Anderssen SA (2006). Physical activity and clustered cardiovascular risk in children: a cross-sectional study (The European Youth Heart Study). The Lancet.

[B29] Dollman J, Norton K, Norton L, Cleland V (2005). Evidence for secular trends in children's physical activity behaviour * Commentary.

[B30] Alexander L, Currie C, Todd J (2003). Gender Matters: Physical activity patterns of schoolchildren in Scotland. Health Behaviour in School-Aged Children.

[B31] Welsh L, Roberts RGD, Kemp JG (2004). Fitness and physical activity in children with asthma. Sports Med.

[B32] Glazebrook C, McPherson AC, Macdonald IAS, Ramsay C, Newbould R, Smyth A (2006). Asthma as a barrier to children's physical activity: implications for body mass index and mental health. Pediatrics.

[B33] Lang DM, Butz AM, Duggan AK, Serwint JR (2004). Physical activity in urban school-aged children with asthma. Pediatrics.

[B34] Meyer A, Machnick MA, Behnke W, Braumann KM (2002). Participation of asthmaic children in gymnastic lessons at school. Pneumologie.

[B35] Sawyer SM, Fardy HJ (2003). Bridging the gap between doctors' and patients' expectations of asthma management. J Asthma.

[B36] Callery P, Milnes L, Verduyn C, Couriel J (2003). Qualitative study of young people's and parents' beliefs about childhood asthma. Br J Gen Pract.

[B37] Chadwick S (1996). The impact of asthma in an inner city general practice. Child Care Health Dev.

[B38] Velsor-Friedrich B, Vlasses F, Moberley J, Coover L (2004). Talking with teens about asthma management. The Journal of School Nursing.

[B39] McCann D, McWhirter J, Coleman H, Devall I, Calvert M, Weare K, Warner J (2002). The prevalence and management of asthma in primary-aged schoolchildren in the south of England. Health Educ Res.

[B40] Clark NM, Partridge MR (2002). Strengthening asthma education to enhance disease control. Chest.

[B41] Fillmore EJ, Jones N, Blankson JM (1997). Achieving treatment goals for schoolchildren with asthma. Arch Dis Child.

[B42] Reading R, Jones T, Upton C (2003). Emergency asthma inhalers in school. Arch Dis Child.

[B43] Lane MM (2006). Advancing the science of perceptual accuracy in pediatric asthma and diabetes. J Pediatr Psychol.

[B44] Male I, Richter H, Seddon P (2000). Children's perception of breathlessness in acute asthma. Arch Dis Child.

[B45] Rietveld S, Brosschot JF (1999). Current perspectives on symptom perception in asthma: a biomedical and psychological review. International Journal of Behavioral Medicine.

[B46] Bandura A (1997). Self-Efficacy: The Exercise of Control.

[B47] Hardeman W, Johnston M, Johnston DW, Bonetti D, Wareham NJ, Kinmonth AL (2002). Application of the Theory of Planned Behaviour Change Interventions:  A Systematic Review. Psychology & Health.

[B48] Conner M, Sparks P, Conner M, Norman P (1998). The theory of planned behaviour and health behaviours. Predicting Health Behaviour.

[B49] Goffman E (1963). Stigma: Notes on the Management of a Spoiled Identity.

[B50] Prout A, Hayes L, Gelder L (1999). Medicines and maintenance of ordinariness in the household management of childhood asthma. Sociology of Health and Illness.

[B51] Davis JM, Watson N (2001). Where are the children's experiences? Analysing social and cultural exclusion in 'special' and 'mainstream' schools. Disability and Society.

[B52] Martel LF, Miller HB (1987). Stature and Stigma: The Biopsychosocial Development of Short males.

[B53] Schmitz KH, Lytle LA, Phillips GA, Murray DM, Birnbaum AS, Kubik MY (2002). Psychosocial correlates of physical activity and sedentary leisure habits in young adolescents: The teens eating for energy and nutrition at school study. Prev Med.

[B54] McQuaid EL, Walders N, Kopel SJ, Fritz GK, Klinnert MD (2005). Pediatric asthma management in the family context: the family asthma management system scale. J Pediatr Psychol.

[B55] Firrincieli V, Keller A, Ehrensberger R, Platts-Mills J, Shufflebarger C, Geldmaker B, Platts-Mills T (2005). Decreased physical activity among head start children with a history of wheezing: Use of an accelerometer to measure activity. Pediatr Pulmonol.

[B56] Lai KY, Lam KKL, Lam SC, Tang ACW, Yeung LKK, Wong MCS, Lee A (2005). Exploring parents' understandings and concerns on self-management of childhood asthma. The Hong Kong Practitioner.

[B57] Bevis M, Taylor B (1990). What do school teachers know about asthma?. Arch Dis Child.

[B58] Neuharth-Pritchett S, Getch YQ (2001). Asthma and the school teacher: The status of teacher preparedness and training. The journal of School Nursing.

[B59] Tse KL, Yu TS (2005). Knowledge of asthma and its management: A study in primary schoolteachers in Hong Kong. The Hong Kong Practitioner.

[B60] Brookes J, Jones K (1992). Schoolteachers' perceptions and knowledge of asthma in primary schoolchildren. Br J Gen Pract.

[B61] Leventhal H, Meyer D, Nerenz D, Rachman S (1980). The common sense representation of illness danger. Medical Psychology.

[B62] Leventhal H, Leventhal EA (2004). Affect, Cognition, and Symptom Perception.

[B63] Weinberger M, Abu-Hasan M (2007). Pseudo-asthma: when cough, wheezing, and dyspnea are not asthma. Pediatrics.

[B64] Cane RS, Ranganathan SC, A`McKenzie S (2000). What do parents of wheezy children understand by "wheeze"?. Archives of Diseases in Childhood.

[B65] World Health Organisation (2003). Adherence to Long-Term Therapies.

[B66] Rietveld S, Beest IV, Everaerd W (1999). Stress-induced breathlessness in asthma. Psychol Med.

[B67] Rietveld S, Everaerd W (2000). Perceptions of asthma by adolescents at home. Chest.

[B68] Chen E, Hermann C, Rodgers D, Oliver-Welker T, Strunk RC (2006). Symptom perception in childhood asthma: the role of anxiety and asthma severity. Health Psychol.

[B69] Melani AS, Ciarleglio G, Pirrelli M, Sestini P (2003). Perception of dyspnea during exercise-induced bronchoconstriction. Respir Med.

[B70] Rietveld S, Everaerd W, Vanbeest I (1999). Can biased symptom perception explain false-alarm choking sensations?. Psychol Med.

[B71] Rietveld S, Prins PJM, Colland VT (2001). Accuracy of symptom perception in asthma and illness severity. Children's Health Care.

[B72] Rietveld S, Kolk AM, Prins PJM (1996). The influence of lung function information on self-reports of dyspnea by children with asthma. J Pediatr Psychol.

[B73] Von-Leupoldt A, Riedel F, Dahme B (2006). The impact of emotions on the perception of dyspnea in pediatric asthma. Psychophysiology.

[B74] Rietveld S, Prins PJM (1998). The relationship between negative emotions and acute subjective and objective symptoms of childhood asthma. Psychol Med.

[B75] Siersted HC, Boldsen J, Hansen HS, Mostgaard G, Hyldebrandt N (1998). Population based study of risk factors for underdiagnosis of asthma in adolescence: Odense schoolchild study. BMJ.

[B76] Coleman H, McCann DC, McWhirter J, Calvert M, Warner JO (2001). Asthma, wheeze and cough in 7- to 9-year-old British schoolchildren. Ambulatory Child Health.

[B77] Lacroix VJ (1999). Exercise-induced asthma. The Physician and Sportsmedicine.

[B78] Michel G, Silverman M, Strippoli MPF, Zwahlen M, Brooke AM, Grigg J, Kuehni CE (2006). Parental understanding of wheeze and its impact on asthma prevalence estimates. Eur Respir J.

[B79] Dowell J, Roberts R, Crombie I, Davis J, Williams B, Skinner J (2007). Reasons for the failure of guideline implementation in primary care: a qualitative case study of epilepsy guidelines. Epilepsia.

[B80] van Sluijs EMF, McMinn AM, Griffin SJ (2007). Effectiveness of interventions to promote physical activity in children and adolescents: systematic review of controlled trials. BMJ.

[B81] Tinkelman D, Schwartz A (2004). School-based asthma disease management. J Asthma.

[B82] Bodenheimer T, Lorig K, Holman H, Grumbach K (2002). Patient self-management of chronic disease in primary care. JAMA.

[B83] Mitchell DK, Murdock KK (2002). Self-competence and coping in urban children with asthma. Children's Health Care.

[B84] Riley KM, Glasgow RE, Eakin EG (2001). Resources for health: A social-ecological intervention for supporting self-management of chronic conditions. Journal of Health Psychology.

[B85] Bailis DS, Fleming JA, Segall A (2005). Self-determination and functional persuasion to encourage physical activity. Psychology and Health.

[B86] Rothman AJ, Salovey P (1997). Shaping perceptions to motivate healthy behavior: the role of message framing. Psychol Bull.

